# Detection of genomic regions underlying resistance to gastrointestinal parasites in Australian sheep

**DOI:** 10.1186/s12711-019-0479-1

**Published:** 2019-07-03

**Authors:** Mohammad Al Kalaldeh, John Gibson, Sang Hong Lee, Cedric Gondro, Julius H. J. van der Werf

**Affiliations:** 1 Cooperative Research Centre for Sheep Industry Innovation, Armidale, NSW 2351 Australia; 2 School of Environmental and Rural Science, University of New England, Armidale, NSW 2351 Australia; 3 Australian Centre for Precision Health, University of South Australia Cancer Research Institute, University of South Australia, Adelaide, SA 5000 Australia; 4 Present Address: College of Agriculture and Natural Resources, Michigan State University, East Lansing, MI 48824 USA

## Abstract

**Background:**

This study aimed at identifying genomic regions that underlie genetic variation of worm egg count, as an indicator trait for parasite resistance in a large population of Australian sheep, which was genotyped with the high-density 600 K Ovine single nucleotide polymorphism array. This study included 7539 sheep from different locations across Australia that underwent a field challenge with mixed gastrointestinal parasite species. Faecal samples were collected and worm egg counts for three strongyle species, i.e. *Teladorsagia circumcincta*, *Haemonchus contortus* and *Trichostrongylus colubriformis* were determined. Data were analysed using genome-wide association studies (GWAS) and regional heritability mapping (RHM).

**Results:**

Both RHM and GWAS detected a region on *Ovis aries* (OAR) chromosome 2 that was highly significantly associated with parasite resistance at a genome-wise false discovery rate of 5%. RHM revealed additional significant regions on OAR6, 18, and 24. Pathway analysis revealed 13 genes within these significant regions (*SH3RF1*, *HERC2*, *MAP3K*, *CYFIP1*, *PTPN1*, *BIN1*, *HERC3*, *HERC5*, *HERC6*, *IBSP*, *SPP1*, *ISG20*, and *DET1*), which have various roles in innate and acquired immune response mechanisms, as well as cytokine signalling. Other genes involved in haemostasis regulation and mucosal defence were also detected, which are important for protection of sheep against invading parasites.

**Conclusions:**

This study identified significant genomic regions on OAR2, 6, 18, and 24 that are associated with parasite resistance in sheep. RHM was more powerful in detecting regions that affect parasite resistance than GWAS. Our results support the hypothesis that parasite resistance is a complex trait and is determined by a large number of genes with small effects, rather than by a few major genes with large effects.

**Electronic supplementary material:**

The online version of this article (10.1186/s12711-019-0479-1) contains supplementary material, which is available to authorized users.

## Background

Gastrointestinal nematode infections (GNI) are one of the most important health problems that affect sheep and other grazing ruminants in Australia and worldwide. The annual cost associated with nematode infections in the Australian sheep industry has been estimated at $436 million for lost production and treatment costs [1]. The effects of parasitism on the health and productivity of grazing ruminants are well documented and include loss of weight, diarrhoea, anorexia, scours, anaemia, and death [2]. During the past few decades, the sheep industry has become increasingly dependent on anthelmintic treatments as a method of parasite control. However, anthelmintic treatments are expensive and often not very effective. The frequent use of these treatments has also resulted in a rapid increase in anthelmintic resistance in sheep worldwide [3, 4]. Breeding sheep for enhanced resistance has been suggested as a viable method of parasite control. The majority of breeding programs for parasite control are based on indicator traits, in particular worm egg count (WEC) in faeces. However, recording WEC is time-consuming, costly, and unattractive. Therefore, it would be useful to select directly for parasite resistance without the need for nematode challenge.

The identification of genes or genomic regions that are responsible for parasite resistance could greatly improve the accuracy of genomic prediction and therefore result in genetic improvement for this trait [5, 6]. Genomic improvement for parasite resistance would benefit from greater knowledge about how sheep are able to mount effective immune responses against parasite infection and the genetic architecture behind the trait. Initial quantitative trait loci (QTL) mapping studies for parasite resistance in sheep were performed using microsatellite markers, e.g. [7–10]. In the last decade, genome-wide association studies (GWAS) using dense single nucleotide polymorphism (SNP) arrays have been used to identify QTL for most of the economically important traits in livestock species. To date, several GWAS have been reported for parasite resistance in different sheep breeds, e.g. [11–14]. Minimal consistency has emerged from these studies, probably due to the physiological complexity of the trait, and the fact that these studies are very diverse in terms of methodologies, statistical approaches, sheep breeds and parasite species.

Genome-wide significant SNPs identified from GWAS for complex traits in sheep, and other species such as humans, have generally failed to explain most of the genetic variation, e.g. [11, 15]. Such studies typically test the association with a phenotype of each SNP individually. In GWAS, the association between each SNP and the trait depends on the existence of linkage disequilibrium (LD) between the observed SNP and the causal loci that underlie the trait. Because of the large number of statistical tests performed in GWAS, very stringent thresholds are applied to avoid spurious associations. These stringent thresholds minimize false positive associations but also lead to many false negatives since variants with small effects or incomplete LD with the SNPs will fail to pass the stringent statistical threshold and remain undetected. Attempts to increase the power of GWAS have focused on increasing the number of observations for each experiment and the density of SNP arrays. Optimizing power in GWAS is both crucial and challenging. Without increasing the number of observations, power could be gained by testing a cumulative effect of multiple variants in a given region of a genome rather than testing each variant individually. Regional heritability mapping (RHM) has been suggested as a better approach to capture more of the genetic variation [16]. RHM facilitates the capture of genetic variation for a given region in the genome by integrating the effects of common and rare variants. Thus, the RHM has the ability to capture some of the genetic variation that is not detected by conventional GWAS methods. The aim of this study was to identify genomic regions with effects on parasite resistance in a large population of sheep naturally challenged with mixed strongyle nematode species (*Teladorsagia circumcincta*, *Haemonchus contortus* and *Trichostrongylus colubriformis*) using both GWAS and RHM approaches.

## Methods

### Phenotypes and population structure

**Fig. 1:**
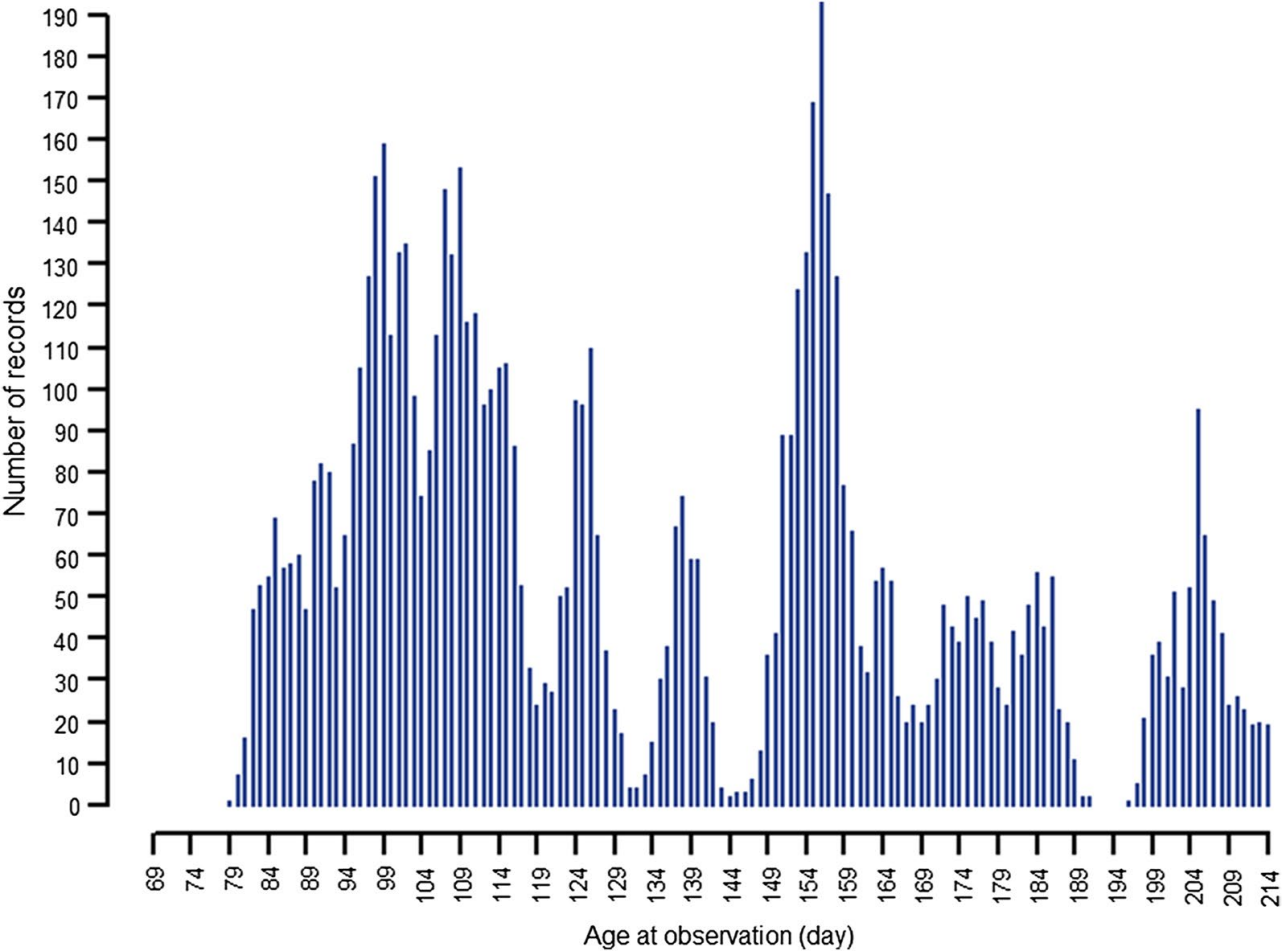
Number of records across ages (per day)

Parasite resistance, as measured by worm egg counts (WEC), was investigated in lambs from a large multi-breed sheep population from the information nucleus (IN) flock of the Australian Sheep Cooperative Research Centre (CRC). Details on the IN flock, design and trait measurements are described in Van der Werf et al. [17]. Lambs were not drenched with anthelmintic until after sampling. When, after weaning, a random faecal sample within a management group exceeded a threshold of 1000 eggs per gram (epg) in sites predominated by *H. contortus*, or 500 epg in sites predominated by other species, faecal samples were collected from all individual animals. Worm eggs were then counted using a modified McMaster counting technique [18]. Worm eggs for three strongyle species were identified, i.e. *T. circumcincta*, *H. contortus* and *T. colubriformis*. The analysis included 7539 animals with both phenotype and genotype data. The distribution of the frequency of WEC records across different ages (days) is shown in Fig. 1.

Various breeds were represented in the population but with a significant proportion of Merino genes (70.0%), and only this breed had a substantial proportion of purebred animals (45.2%). The remaining breeds were represented mainly by crossbred offspring of their sires in crosses with Merino or Border Leicester × Merino ewes. Breed group size ranged from 3493 sheep for purebred Merino to 97 for a Poll Dorset/Suffolk/White Suffolk/White Dorper/Border Leicester/Merino cross. The breed content of the population is in Table 1.

**Table 1:** Proportions of different breeds’ ancestry in the population

Breed	BL	COR	SUF	WS	BRL	WD	PD	TEX	PS	MER
Proportion (%)	10.9	0.8	2.5	1.7	0.7	0.4	10.0	1.8	1.2	70.0

*BL* Border Leicester, *COR* Corriedale, *WD* White Dorper, *PD* Poll Dorset, *TEX* Texel, *PS* Prime Samm, *MER* Merino, *SUF* Suffolk, *WS* White Suffolk, *BRL* Booroola

### Genotypes and quality controls

#### 50 k genotypes

Animals were genotyped with the Illumina 50 K SNP panel (Illumina Inc., San Diego, CA, USA). Several quality measures were applied to the 50 k SNP data. SNPs were removed if they had a minor allele frequency (MAF) lower than 0.01, a call rate lower than 90%, an Illumina Gentrain score (GC) lower than 0.6, a *p* value testing Hardy–Weinberg equilibrium less than 10^−15^, if the heterozygosity rate deviated by more than 3 standard deviations from the population mean and if they were located on the X and Y chromosomes. Furthermore, an individual was removed if the correlation of the genotypes with another sample (animal) was equal or higher than 0.99. After applying the quality control measures, 48,599 SNPs were retained for the analyses.

#### High-density (HD) genotypes

All animals with WEC phenotypes were then imputed from 50 K genotypes to the 600kOvine Infinium^®^ HD SNP BeadChip panel (International Sheep Genomic Consortium and FarmIQ Project NZ). The high-density (HD) genotypes were imputed using a reference set of 1881 animals with real HD genotypes. This reference set of HD genotyped animals represented four main breeds (Merino, Poll Dorset, Border Leicester, and White Suffolk): 1042 represented various crosses of these breeds, while purebreds included 677 Merino, 47 White Suffolk, 44 Poll Dorset, 32 Border Leicester, and 39 from 10 other breeds. After applying the same quality measures as above, 510,065 SNPs were retained, and these 1881 HD animals were then used as a reference set to impute the 50 K genotypes to HD using Minimac3 [19]. Prior to imputation, phasing was performed on both the 50 K-genotyped and HD-genotyped animals separately using Eagle2 [20]. The accuracy of imputation to HD, which was tested within subsets of animals with observed HD genotypes, was on average high (0.98) across the whole genome.

### Genome-wide association studies (GWAS)

In order to reduce computation time, a two-step association analysis was performed. First, phenotypes were pre-adjusted for fixed effects using the following model:

1 $${\mathbf{y}} = {\mathbf{1}} \mu + {\mathbf{Xb}} + {\mathbf{e}} ,$$

where ${\mathbf{y}}$ is a vector of cube-root transformed WEC records; $\varvec{ }\mu$ is the overall mean; ${\mathbf{X}}$ is a design matrix of fixed effects; ${\mathbf{b}}$ is a vector of fixed effects and ${\mathbf{e}}$ is a vector of residuals assumed to be distributed as $\sim N\left( {0,{\mathbf{I}}\sigma_{e}^{2} } \right)$, where ${\mathbf{I}}$ is the identity matrix and $\sigma_{e}^{2}$ is the residual variance. The fixed effects included in the models to determine the corrected phenotypes were age of animals at WEC recording, age of dam, gender, rearing type $\times$ birth type, contemporary groups (combination of flock, birth year and management group effects) and breed composition, which were fitted as covariates, one for each contributing breed. Second, residuals obtained from Model 1 were treated as corrected phenotypes for a single-SNP regression where each SNP was fitted separately, and a pedigree relationship matrix was fitted to account for population and pedigree structure. A linear mixed model was performed using the GEMMA program [21] as follows:

2 $${\mathbf{y}}^{*} = {\mathbf{1}}\mu + {\mathbf{W}}_{{\mathbf{i}}} g_{i} + {\mathbf{Za}} + {\mathbf{e}} ,$$

where ${\mathbf{y}}^{*}$ is a vector of adjusted phenotypes (residuals) obtained from Eq. 1, $\varvec{ }\mu$ is the overall mean, ${\mathbf{W}}_{i}$ is a vector of genotypes for ${\text{SNP}}_{i}$ (coded as 0, 1, or 2 for the genotypes 00, 01/10, or 11, respectively), $g_{i}$ is the effect size of the $i$th SNP (allele substitution effect), ${\mathbf{Z}}$ is a design matrix of random additive genetic effects, ${\mathbf{a}}$ is a vector of random additive genetic effects assumed to be distributed as $\sim N\left( {0,{\mathbf{A}}\sigma_{a}^{2} } \right)$, where ${\mathbf{A}}$ is the numerator relationship matrix calculated from available pedigree using the pedigree package in R [22], and ${\mathbf{e}}$ is the vector of residuals. The false discovery rate (FDR) was applied to adjust for multiple SNP testing. Significant SNPs were determined by using the genome-wise FDR of 5%.

### Regional heritability mapping (RHM)

RHM analyses were carried out on the whole genome using the MTG2 software [23]. Each chromosome was divided into regions that contained a predefined number of SNPs, and the additive genetic variance attributable to the joint SNP effects within each window was estimated. Window sizes of 1000 SNPs (~ 5 Mbp), 500 SNPs (~ 2.5 Mbp) and 200 SNPs (~ 1 Mbp) were used to build the genomic relationship matrices (GRM) for the specific regions and the windows were then shifted along the genome in steps of 500, 250 and 100 SNPs, respectively. To test the significance of each window, a likelihood ratio test (LRT) was applied to compare the full model, which includes the regional effect (Eq. 3) with the reduced model with no regional variance in that window (Eq. 4):

3 $${\mathbf{y}}^{*} = {\mathbf{1}}\mu + {\mathbf{Z}}_{{\mathbf{i}}} g_{i} + {\mathbf{Za}} + {\mathbf{e}} ,$$

4 $${\mathbf{y}}^{*} = {\mathbf{1}}\mu + {\mathbf{Za}} + {\mathbf{e}} ,$$

where the terms are as defined in Eqs. 1 and 2, except $g_{i}$, which is the additive genetic effect of the window genotype estimated from SNPs within region $i$ and assumed to be distributed as $N\left( {0,{\mathbf{GRM}}_{i} \sigma_{{g_{i} }}^{2} } \right)$, where ${\mathbf{GRM}}_{i}$ is the regional genomic relationship matrix constructed from SNPs within region $i$, and $\sigma_{{g_{i} }}^{2}$ is the genomic variance explained by the SNPs in region $i$. Phenotypic variance was given by $\sigma_{p}^{2} = \sigma_{{g_{i} }}^{2} + \sigma_{a}^{2} + \sigma_{e}^{2}$ and therefore the regional genomic heritability was estimated as ${\text{h}}_{{g_{i} }}^{2} = \sigma_{{g_{i} }}^{2} /\sigma_{p}^{2}$.

For the RHM approach, LRT was assumed to follow a mixture of $0.5 \chi_{\left( 1 \right)}^{2}$ and $0.5 \chi_{\left( 0 \right)}^{2}$ distributions [16]. In total, 980, 2005 and 5025 windows were tested across the genome using RHM with window sizes of 1000, 500 and 200 SNPs, respectively. Due to the large number of windows tested across the genome, FDR was applied to correct for multiple testing. Significant windows were selected by using the genome-wise FDR of 5%.

### Conditional GWAS and RHM analyses

Conditional GWAS and RHM analyses were carried out to determine if significant SNPs and regions were independent. First, GWAS analyses were performed on the regions of *Ovis aries* (OAR) chromosomes 2 and 6 by including the most significant SNP as a fixed covariate in the model and testing all SNPs in the region that were not in strong LD with the conditional SNP ($r^{2} < 0.95$). Second, RHM analyses were performed by adding the most significant regions to the model in a stepwise manner. To obtain a more conservative and probably better heritability estimate for a given region, both ${\mathbf{GRM}}_{i}$ from the significant regions, and its complementary GRM (${\mathbf{GRM}}_{c}$), which is based on all the remaining SNPs in the 600 K SNP panel, were fitted jointly in one model. Significant regions were added to the model sequentially where, each time, a new ${\mathbf{GRM}}_{i}$ was built from all regions combined and a new ${\mathbf{GRM}}_{c}$ was also built from all the SNPs on the 600 K panel, excluding the unique fitted SNPs in ${\mathbf{GRM}}_{i}$. Third, RHM analyses were performed conditionally on the top SNP from each region, which were then added to the model sequentially as fixed covariates, and the proportion of variance explained by all regions combined was estimated.

### Haplotype construction and analysis

Haplotypes were constructed for SNPs located within the statistically significant windows using the Fimpute algorithm [24]. Once the haplotypes were constructed, LD between SNPs was calculated as the D′ statistics using the Haploview software [25], with haplotype blocks defined based on the criteria of Gabriel et al. [26]. Haplotype analysis was then carried out using the following model:

5 $${\mathbf{y}}_{{\mathbf{j}}}^{*} = \mu + \mathop \sum \limits_{i = 1}^{t} \beta_{ij} H_{i} + {\mathbf{a}}_{{\mathbf{j}}} + {\mathbf{e}}_{{\mathbf{j}}} ,$$

where ${\mathbf{y}}_{{\mathbf{j}}}^{*}$ is the adjusted phenotypic (residual) value for the $j$th animal; $\mu$ is the overall mean; $H_{i}$ is the effect of the $i$th haplotype; $\beta_{ij}$ is the haplotype score (0, 1, or 2) of the $i{\text{th}}$ haplotype for the $j{\text{th}}$ animal, $t$ is the number of haplotypes segregating in the population for that haplotype block; ${\mathbf{a}}_{j}$ is the vector of random additive genetic effects of individual $j$ and ${\mathbf{e}}_{j}$ is the vector of random residual effects.

### Principal component analysis

Principal component analysis (PCA) was performed on the genomic relationship matrix using MTG2. The first two principal components (PC) of the genotyped animals were plotted and samples were coloured according to their breed compositions, as known from pedigree information.

### Gene annotation and functional information

Candidate genes in the significant regions were obtained from Ensembl (http://www.ensembl.org/biomart) and UCSC Genome Bioinformatics (http://genome.ucsc.edu). Because the sheep genome is not completely annotated compared to the human genome, human orthologous genes were used to explore their molecular functions. Biological pathways associated with these identified genes were obtained using the BioSystem Tools (https://www.ncbi.nlm.nih.gov/biosystems), which contains pathways from the main databases including: KEGG (Kyoto Encyclopedia of Genes and Genomes), Pathway Interaction Database (PID), WikiPathways and Reactome.

## Results

### Association analyses

**Fig. 2:**
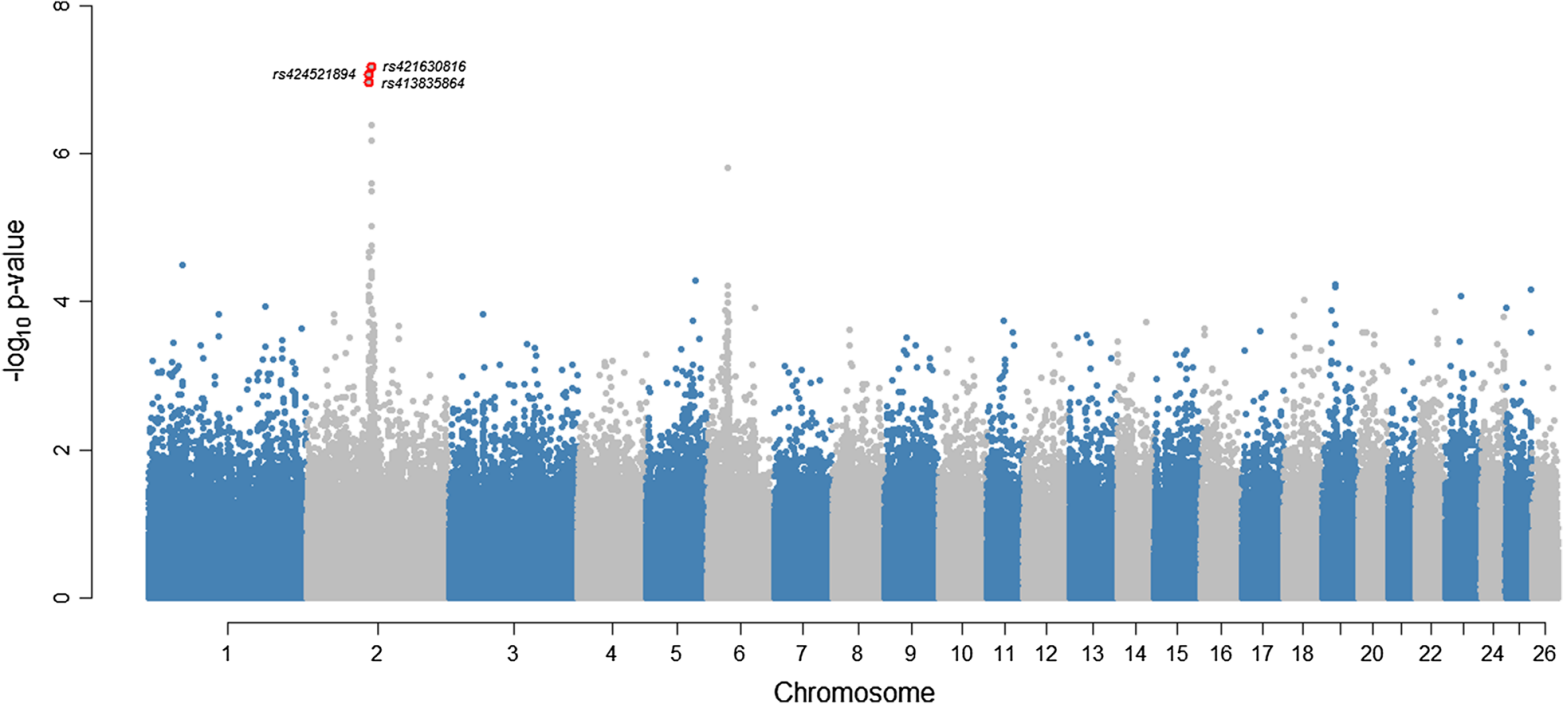
Manhattan plot of GWAS results for parasite resistance in Australian sheep. The y-axis shows the $- log_{10}$ (*p*-values) of single-SNP association and the x-axis shows the position of the SNPs across the 26 chromosomes. Genome-wide significant SNPs (FDR of 5%) are highlighted by red dots

**Fig. 3:**
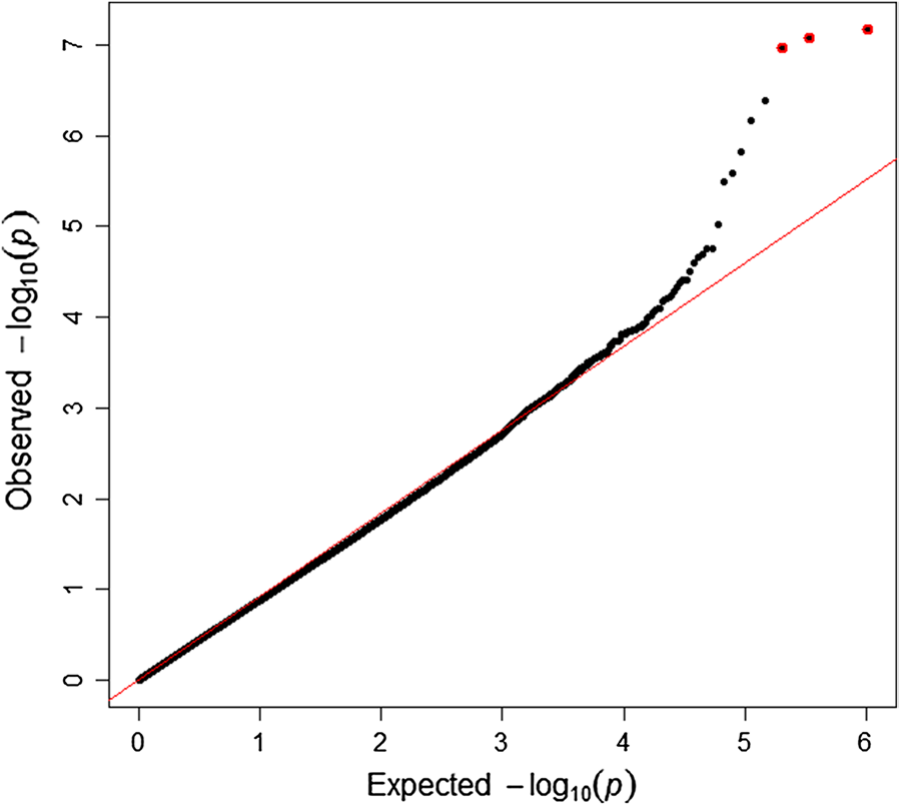
Quantile-quantile (Q–Q) plot of the observed *p*-values from the expected *p*-values of GWAS results (λ = 0.95). The observed $- log_{10}$ (*p*-values) are shown by black dots, and the expected values under null distribution are shown by a red line. Significant SNPs (FDR of 5%) are highlighted by red dots

A Manhattan plot of GWAS results for parasite resistance in sheep is in Fig. 2. Three SNPs (rs421630816, rs424521894, and rs413835864) were statistically significant at a genome-wise FDR of 5% (Table 2). The quantile–quantile (Q–Q) plot shows that, for these three significant SNPs, the deviation from their expected values is larger, which indicates a strong association between these SNPs and parasite resistance (Fig. 3). SNP rs421630816 is located within the *PALLD* gene at 110.8 Mbp on OAR2, and the rs424521894 and rs413835864 SNPs are located within the *GALNTL6* gene at 107.3 and 107.4 Mbp on OAR2, respectively.

**Table 2:** List of significant SNPs identified by GWAS for parasite resistance in Australian sheep

OAR	SNP	Variant type	Position (chr:bp)	Nearest gene		p-value	FDR
				Name	Distance		
2	rs421630816	Intron	2:110875234	*PALLD*	Within	6.7 × 10^−8^	0.02
2	rs424521894	Downstream gene	2:107301187	*GALNTL6*	Within	8.6 × 10^−8^	0.02
2	rs413835864	Intron	2:107458856	*GALNTL6*	Within	1.1 × 10^−7^	0.03

Significant SNPs were selected at a FDR of 5%

**Fig. 4:**
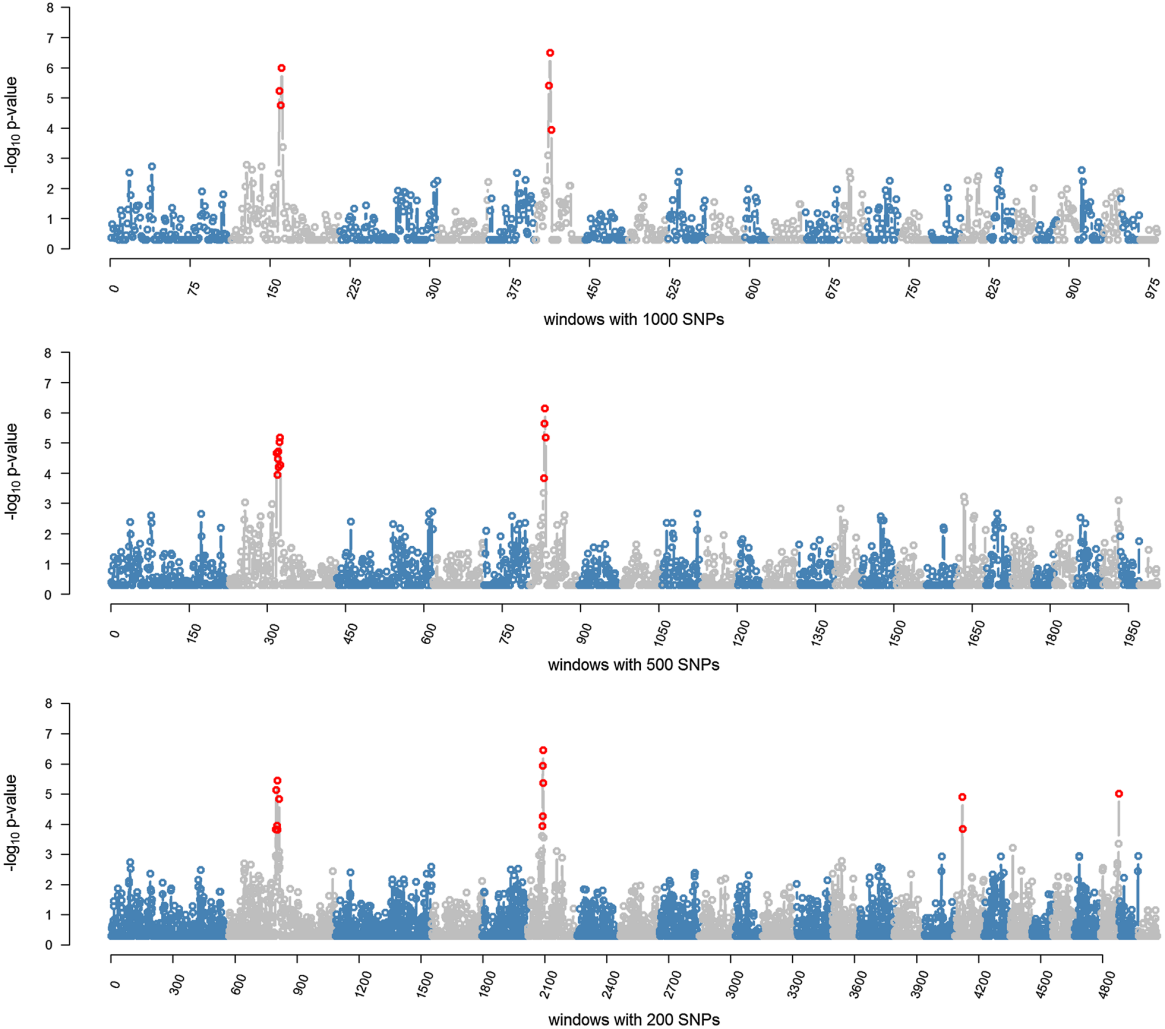
Regional heritability mapping (RHM) across the genome. The y-axis shows the $- log_{10} \left( {p - value} \right)$ associated with each window and the x-axis shows the window number across the genome. Windows were analyzed using 1000-SNP (top plot), 500-SNP (middle plot) and 200-SNP windows (lower plot). Genome-wide significant windows (FDR of 5%) are highlighted by red dots

**Fig. 5:**
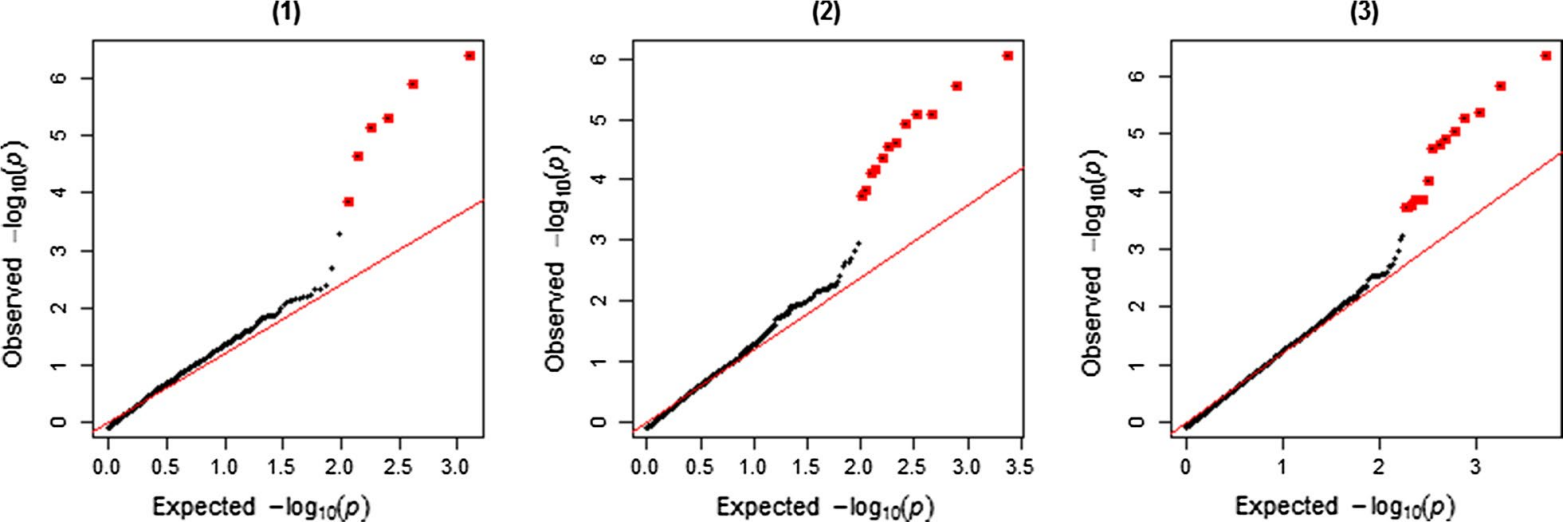
Quantile-quantile (Q–Q) plot of the observed *p*-values from the expected *p*-values of RHM using (1) 1000-SNP window size (λ = 1.06), (2) 500-SNP window size (λ = 1.08), and 200-SNP window size (λ = 1.09). The observed $- log_{10}$ (*p*-values) are shown by black dots, and the expected values under null distribution are shown by a red line. Significant windows (FDR of 5%) are highlighted by red dots

The results from the RHM analyses using window sizes of 1000, 500 and 200 SNPs are in Table 3 and Fig. 4. Q–Q plots of observed versus expected *p*-values of RHM analyses are in Fig. 5. RHM analysis using 1000-SNP windows identified three overlapping windows between 106.4 and 118.7 Mbp on OAR2, and another three overlapping windows between 32.7 and 42.3 Mbp on OAR6 that were significantly associated with parasite resistance at the genome-wide level. RHM analysis with 200-SNP windows identified three regions on OAR2: two overlapping windows between 106.9 and 108.4 Mbp, three overlapping windows between 110.1 and 113.3 Mbp, and one window between 117.01 and 118.13 Mbp. The two overlapping windows between 106.9 and 108.4 Mbp on OAR2 contains the second and third most significant SNPs (rs413835864 and rs424521894) identified by the GWAS, whereas the window between 110.59 and 112.38 Mbp contains the top significant SNP (rs421630816). Fine-mapping analysis also identified a significant region between 34.7 and 39.2 Mbp on OAR6, which included the sixth top ranked SNP (rs416517011; *p* = 7.87 × 10^−8^) based on GWAS. In addition, RHM using 200-SNP windows identified a region that contains two overlapping windows between 17.6 and 18.9 Mbp on OAR18, and a region on OAR24 between 40.4 and 41.9 Mbp.

**Table 3:** Summary of significant windows identified by RHM using window sizes of 1000, 500 and 200 SNPs

Window #	OAR	Position (bp)		${\text{h}}_{{g_{i} }}^{2}$ (SE)	LRT	−logP	FDR
		Start	End				
1000-SNP window analysis							
159	2	106479658	112388825	0.007 $\left( {0.003} \right)$	19.2	5.23	0.001
160	2	109055156	115988999	0.007 $\left( {0.004} \right)$	17.2	4.76	0.003
161	2	112409311	118712363	0.010 $\left( {0.005} \right)$	22.6	5.99	0.001
412	6	32700889	37820042	0.009 $\left( {0.004} \right)$	20.0	5.40	0.001
413	6	35275766	40133729	0.010 $\left( {0.004} \right)$	24.8	6.49	0.000
414	6	37828214	42360019	0.007 $\left( {0.004} \right)$	13.6	3.93	0.019
500-SNP window analysis							
318	2	106479658	109049182	0.006 $\left( {0.003} \right)$	16.7	4.66	0.006
319	2	107706511	110358002	0.007 $\left( {0.004} \right)$	13.6	3.93	0.021
320	2	109055156	112388825	0.007 $\left( {0.004} \right)$	15.9	4.46	0.009
321	2	110360839	114468943	0.006 $\left( {0.003} \right)$	17.0	4.71	0.006
322	2	112409311	115988999	0.007 $\left( {0.004} \right)$	14.7	4.19	0.013
323	2	114470485	117276436	0.012 $\left( {0.007} \right)$	18.3	5.02	0.004
324	2	115995299	118712363	0.008 $\left( {0.004} \right)$	18.9	5.17	0.003
325	2	117294302	119911849	0.006 $\left( {0.003} \right)$	15.0	4.26	0.012
830	6	34069188	36548480	0.007 $\left( {0.004} \right)$	13.1	3.82	0.025
831	6	35275766	37820042	0.009 $\left( {0.005} \right)$	21.0	5.64	0.002
832	6	36552018	38994028	0.007 $\left( {0.003} \right)$	23.2	6.14	0.001
833	6	37828214	40133729	0.008 $\left( {0.004} \right)$	19.0	5.18	0.003
200-SNP window analysis							
798	2	106987063	107924749	0.005 $\left( {0.003} \right)$	13.13	3.83	0.051
799	2	107447474	108441185	0.007 $\left( {0.003} \right)$	18.8	5.14	0.007
804	2	110107622	111043903	0.007 $\left( {0.004} \right)$	13.6	3.95	0.048
805	2	110590464	112388825	0.006 $\left( {0.003} \right)$	20.2	5.46	0.005
806	2	111047822	113380103	0.006 $\left( {0.004} \right)$	13.1	3.82	0.055
814	2	117019488	118134501	0.007 $\left( {0.004} \right)$	17.5	4.83	0.009
2088	6	35275766	36251932	0.005 $\left( {0.003} \right)$	13.6	3.95	0.047
2089	6	35743335	36802158	0.005 $\left( {0.003} \right)$	13.5	3.95	0.047
2090	6	36252650	37275709	0.005 $\left( {0.003} \right)$	15.0	4.27	0.030
2091	6	36805051	37820042	0.010 $\left( {0.006} \right)$	22.4	5.94	0.003
2092	6	37286366	38279284	0.008 $\left( {0.004} \right)$	24.6	6.45	0.002
2093	6	37828214	38766020	0.006 $\left( {0.003} \right)$	19.8	5.36	0.005
4122	18	17645871	18425906	0.007 $\left( {0.003} \right)$	17.8	4.91	0.009
4123	18	18057085	18957804	0.005 $\left( {0.003} \right)$	13.2	3.84	0.051
4879	24	40476812	41995998	0.008 $\left( {0.004} \right)$	18.3	5.02	0.008

${\text{h}}_{{g_{i} }}^{2}$: regional genomic heritability; SE: standard error; -logP: $- { \log }_{10} \left( {{\text{p}} - {\text{value}}} \right)$

Significant windows were selected at a FDR of 5%

**Fig. 6:**
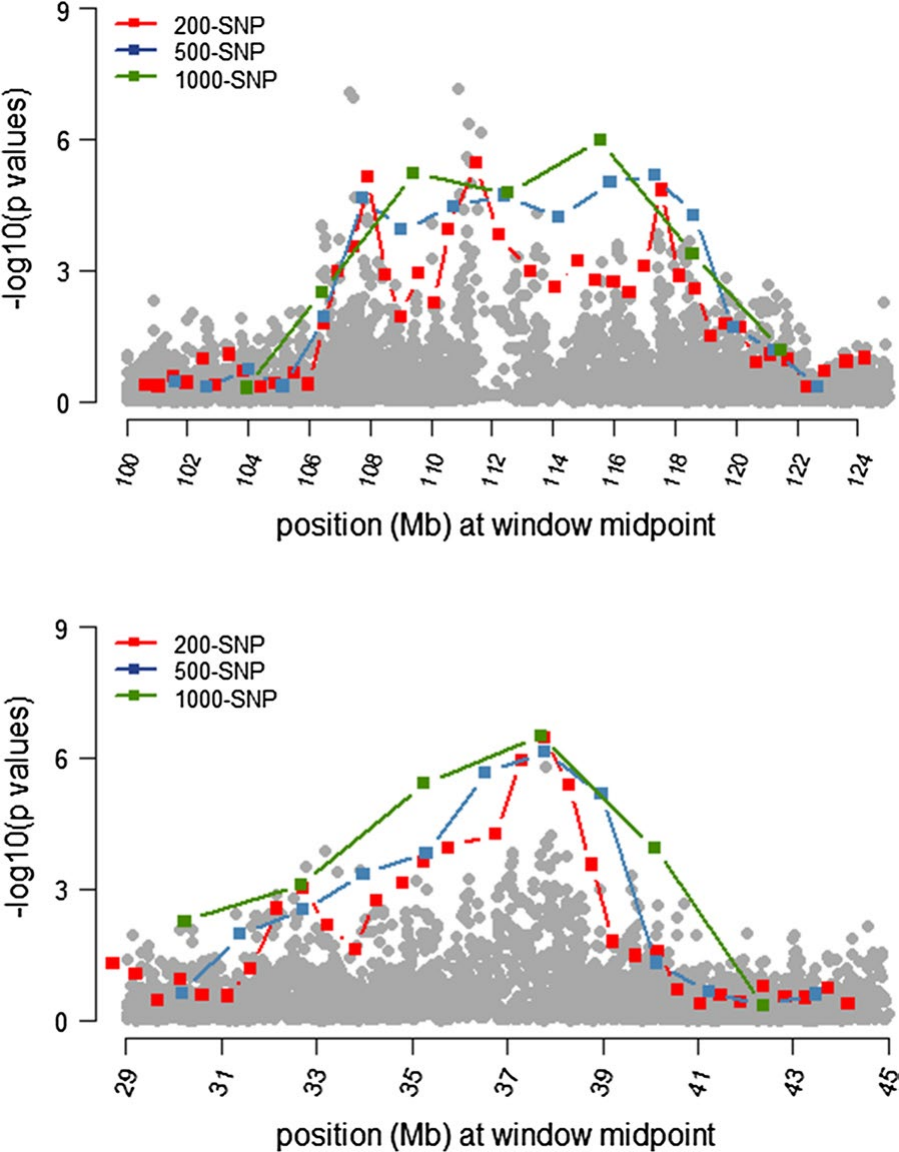
RHM and GWAS results of the identified region on OAR2 between 100 and 124 Mb (top plot) and on OAR6 between 28 and 44 Mb (bottom plot). The solid lines show the RHM results using three window sizes (1000 SNPs = green; 500 SNPs = blue; 200 SNPs = red), where each window is positioned at the window midpoint. Grey dots show the GWAS results within each region

Figure 6 shows a comparison of the RHM results among the three window sizes used (1000-SNP, 500-SNP and 200-SNP) for the target regions on OAR2 and OAR6. The 200-SNP window RHM analysis improved the mapping resolution of the identified regions (regions became narrower). However, the overall power was not higher compared to the larger window sizes since the LRT values did not increase when window sizes were shifted from 1000 to 200 SNPs.

**Fig. 7:**
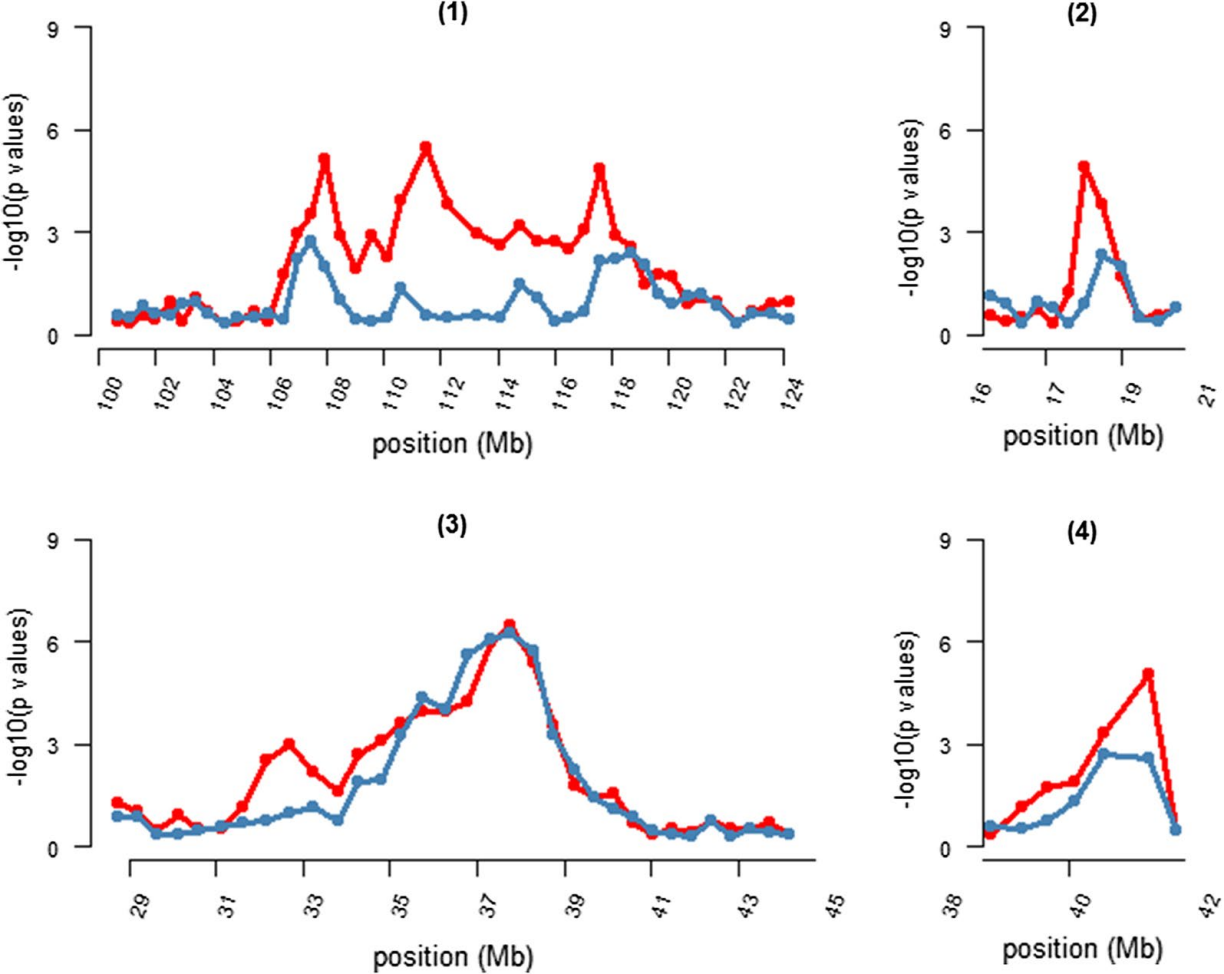
Comparison of RHM results using 200-SNP windows between the Merino population and the entire sheep population for the target regions on (1) OAR2, (2) OAR18, (3) OAR6, and (4) OAR24. The red lines represent the RHM results for the entire sheep population and blue lines represent the RHM results for Merino sheep population

The significant regions identified by RHM were re-analysed using 200-SNP windows for Merino sheep only to validate whether the same target regions persisted within the Merino sheep population. The results show that the significance of the peaks for the target regions on OAR2, 18, and 24 decreased, whereas the peak in the OAR6 region was maintained (Fig. 7). These results are likely due to the reduced sample size, with half the animals being Merino sheep, which resulted in the statistical power being inadequate to confirm the association in these target regions.

### Conditional association analyses

**Fig. 8:**
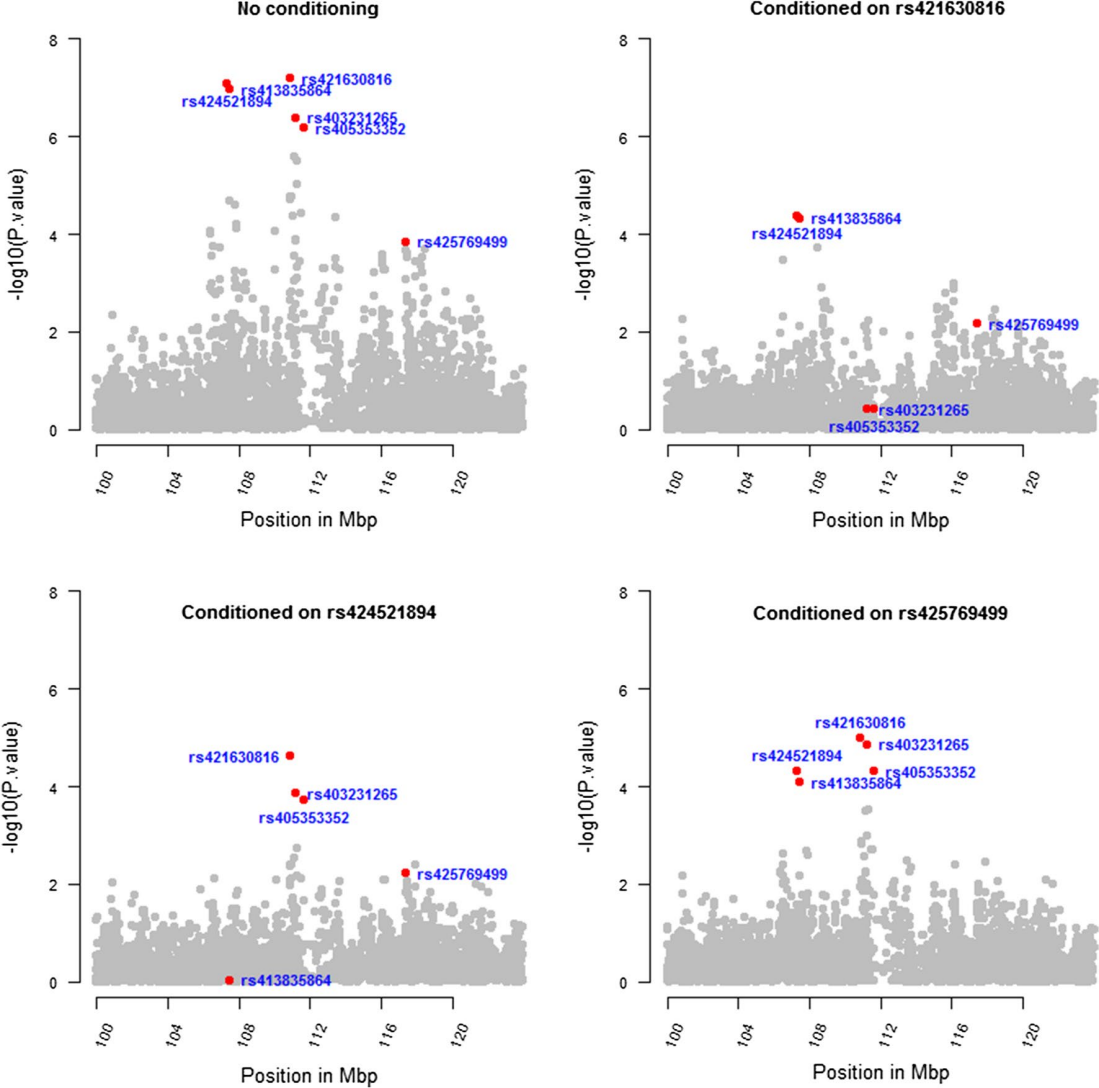
Conditional GWAS plots for the region between 100 and 120 Mbp on OAR2. The first plot shows GWAS results with no conditioning. Subsequent plots show GWAS results conditioned on the first and second significant SNPs (rs421630816 and rs424521894) as well as SNP rs425769499, the top ranked SNP in a region between 117 and 118 Mbp

**Fig. 9:**
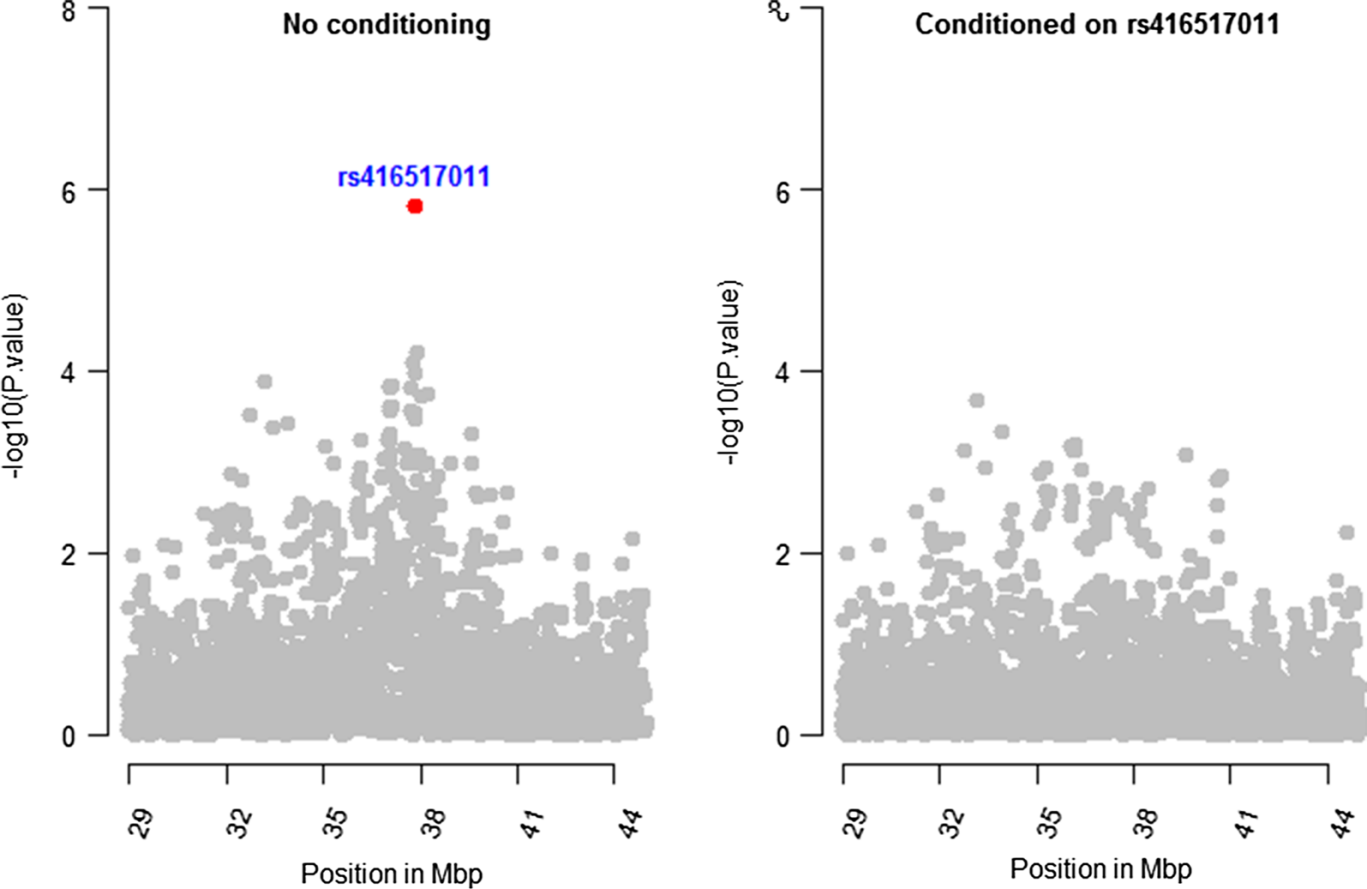
Conditional GWAS plots for the region between 29 and 44 Mbp on OAR6. The left plot shows GWAS results with no conditioning. Right plot shows GWAS results conditioned on rs416517011, the sixth top ranked SNP based on GWAS

Results of conditional GWAS analyses for the significant regions on OAR2 and 6 are shown in Figs. 8 and 9, respectively. GWAS analysis conditioned on the first top significant SNP (rs421630816) removed the peaks in the region between 110 and 112 Mbp on OAR2. The *p*-values of the fourth and fifth top ranked SNPs (rs403231265 SNP; *p* = 4.2 × 10^−7^ and rs405353352 SNP; *p* = 6.7 × 10^−7^) at 111 Mbp on OAR2 became 0.38, whereas the *p*-values for the second and third significant SNPs (rs424521894 and rs413835864) at 107 Mbp remained lower than 10^−4^. When GWAS was conditioned on rs424521894 SNP, the *p*-value for rs413835864 became 0.9, whereas *p*-values for rs421630816 remained lower than 2 × 10^−5^. GWAS analysis conditioned on rs425769499 SNP, which is the top ranked SNP in the region between 117 and 118 Mbp, was also performed and the *p*-values for all significant SNPs on OAR2 remained lower than 10^−4^. LD between rs424521894, rs421630816, and rs425769499, i.e. the top SNP in each of the three regions between 107 and 108, 110 and 112, and 117 and 118 Mbp, was zero for all pairwise comparisons. However, LD between rs424521894 and rs413835864 SNPs of the same region was moderate ($r^{2} = 0.41$), whereas LD between rs421630816 SNP and either of the rs403231265 and rs405353352 SNPs of the same region was moderate ($r^{2} = 0.26$) and strong ($r^{2} = 0.86$), respectively.

The proportion of phenotypic variance explained by the significant regions gradually increased with each additional region fitted in the model (Table 4), ranging from 0.004 (s.e. = 0.002) by fitting only the region between 107 and 108 Mbp on OAR2 to 0.030 (s.e. = 0.008) by fitting all five significant regions combined in one GRM. The model fit, as measured by LRT, gradually improved with each additional region fitted in the model.

**Table 4:** RHM analysis conditioned on adding significant regions to the model sequentially

Scenario	${\mathbf{GRM}}_{i}$ (SE)	${\mathbf{GRM}}_{c}$ (SE)	Logl	Logl null	LRT
R1	0.004 (0.002)	0.19 (0.02)	− 10667	− 10671	8
R1 + R2	0.006 (0.003)	0.18 (0.02)	− 10666	− 10672	12
R1 + R2 + R3	0.008 (0.003)	0.18 (0.02)	− 10664	− 10672	16
R1 + R2 + R3 + R4	0.015 (0.005)	0.17 (0.02)	− 10656	− 10674	36
R1 + R2 + R3 + R4 + R5	0.024 (0.007)	0.16 (0.02)	− 10649	− 10674	50
R1 + R2 + R3 + R4 + R5 + R6	0.030 (0.008)	0.16 (0.02)	− 10644	− 10675	62

R1: between 107 and 108 Mbp on OAR2; R2: between 110 and 113 Mbp on OAR2; R3: between 117 and 118 Mbp on OAR2; R4: between 34 and 39 Mbp on OAR6, R5: between 17 and 18 Mbp on OAR18; and R6: between 40 and 41 Mbp on OAR24

${\mathbf{GRM}}_{i}$: variance due to regions defined in each scenario and estimated with a GRM constructed from SNPs in these regions

${\mathbf{GRM}}_{c}$: is the complementary GRM containing all SNPs from the 600 k excluding the SNPs fitted in ${\mathbf{GRM}}_{i}$

Logl: log likelihood for the tested model which includes both ${\mathbf{GRM}}_{i}$ and ${\mathbf{GRM}}_{c}$

Logl null: log likelihood for the null model which includes only ${\mathbf{GRM}}_{c}$

SE: standard error

The proportion of phenotypic variance explained by all combined regions gradually decreased from 0.030 to 0.028 when RHM was conditioned only on the rs421630816 SNP, and from 0.030 to 0.019 when RHM was conditioned on the top six SNPs (Table 5). The model fit also improved with each additional SNP fitted as a fixed covariate except when the rs425769499 SNP was included.

**Table 5:** RHM analysis conditioned on adding the top SNPs from GWAS to the model sequentially

Scenario	${\mathbf{GRM}}_{i}$ (SE)	${\mathbf{GRM}}_{c}$ (SE)	Logl	LRT
No condition	0.030 (0.008)	0.16 (0.02)	− 10644	–
Cond. on S1	0.028 (0.008)	0.16 (0.02)	− 10643	2
Cond. on S1 + S2	0.026 (0.007)	0.16 (0.02)	− 10642	4
Cond. on S1 + S2 + S3	0.026 (0.007)	0.16 (0.02)	− 10644	0
Cond. on S1 + S2 + S3 + S4	0.023 (0.007)	0.16 (0.02)	− 10641	6
Cond. on S1 + S2 + S3 + S4 + S5	0.020 (0.006)	0.16 (0.02)	− 10637	14
Cond. on S1 + S2 + S3 + S4 + S5 + S6	0.019 (0.006)	0.16 (0.02)	− 10636	16

**GRM**_**i**_ and **GRM**_**c**_ as in Table 4

S1: rs421630816; S2: rs424521894; S3: rs425769499; S4: rs416517011; S5: rs404837788; S6: rs413573644

Logl: log likelihood

SE: standard error

### Haplotype analysis

**Fig. 10:**
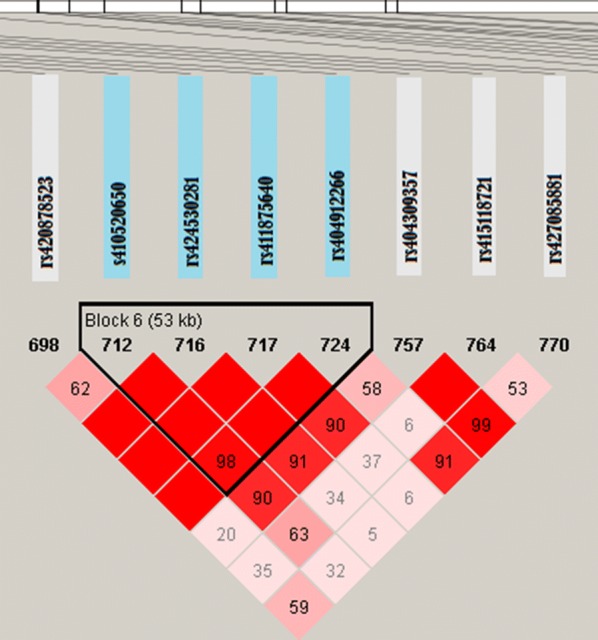
Linkage disequilibrium (LD) map for the region between 107.33 and 107.38 Mbp on OAR2. The haplotype block (block 6) containing the four SNPs (rs410520650, rs424530281, rs411875640 and rs404912266) is highlithed in blue boxes

Haplotype analysis was performed for the region between 106.4 and 118.7 Mbp on OAR2 and the region between 32 and 36 Mbp on OAR6. Using the criteria described by Gabriel et al. [26], SNPs that showed high LD within each region were grouped together in haplotype blocks. Twenty-six and 10 haplotype blocks were identified in the regions on OAR2 and 6, respectively. Haplotype block sizes ranged from 2 to 141 kb. Using Eq. 5, only block 6 (Fig. 10) located between 107.33 and 107.38 Mbp on OAR2 had a significant effect on parasite resistance (*p*-value = 0.003). Six distinct haplotypes were identified in this haplotype block (Table 6). Haplotypes TTTG and CTTA had positive significant effects on parasite resistance, while haplotype CTTG had a marginally negative effect.

**Table 6:** Haplotype association analysis of block 6 (107.33–107.38 Mbp on OAR2) with parasite resistance

Haplotype	Effects^a^ ($\beta$)	$p$-value
TTTG	0.093388	0.00601
TTTA	0.002921	0.89287
CTTG	− 0.083304	0.02507
CATG	− 0.045274	0.83761
CACG	− 0.028541	0.39638
CTTA	0.816840	0.00592

The overall association between haplotypes and the trait was significant ($p$ < 0.003). Significance level at 0.05.

^a^Estimates of regression coefficients ($\beta$) in phenotypic standard deviation (STD) units of the trait

^2^The $p$ for testing null hypothesis of $\beta = 0$

### Principal components

**Fig. 11:**
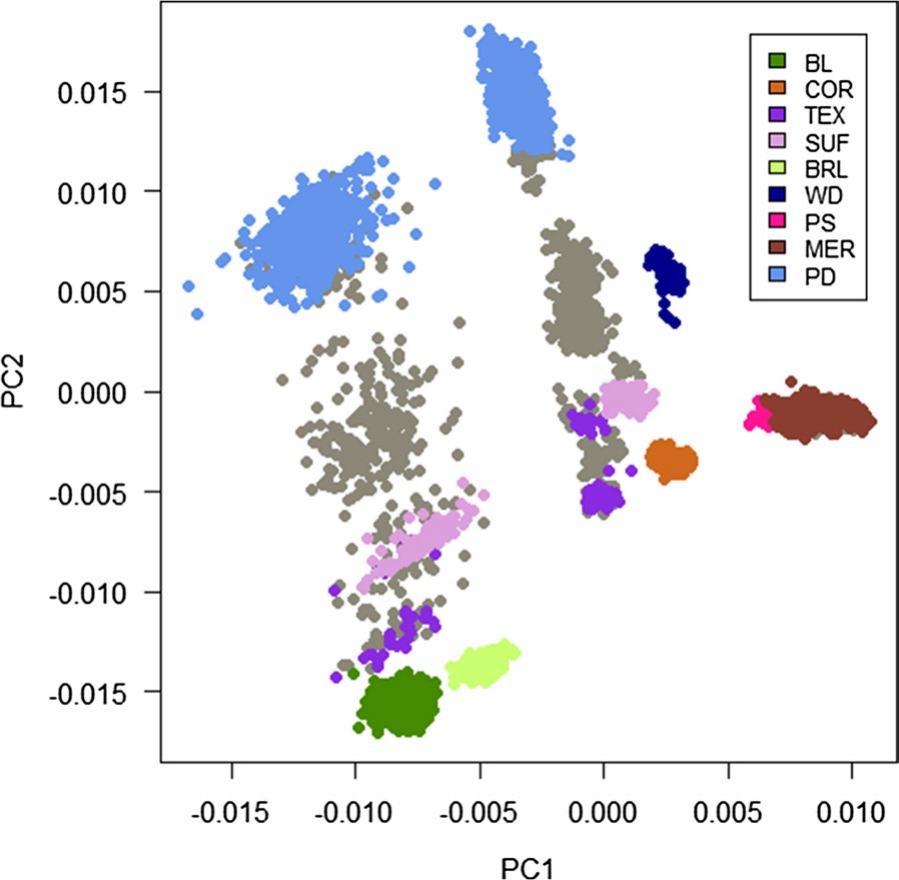
Clustering of animals based on the plot of principal components (PC1 and PC2). Animals were coloured based on their breed composition of purebred Merino and composition of other breeds, with the breed that makes up more than 50% of the breed proportion. *BL* Border Leicester; *COR* Corriedale; *TEX* Texel; *SUF* Suffolk; *BRL* Booroola; *WD* White Dorper; *PS* Prime Samm; *PD* Poll Dorset; and *MER* pure Merino. The grey colour represents animals of different crosses but with less than 50% of a particular breed composition

The first two principal components (PC1 and PC2) are plotted and annotated by breed composition of the animals (Fig. 11). Only Purebred Merino and other breeds that make up 50% and more of the animals breed composition were annotated.

Pathway analysis was performed to link genes within the significant regions on OAR2, 6, 18 and 24 to their biological pathways using the BioSystem Tools from NCBI. The analysis linked 84 genes (Table 7) to 271 unique pathways (see Additional file 1: Table S1). The number of genes in the pathways ranged from 1 to 10 genes, with a median size of 1. Pathways with the largest sizes were ‘Metabolism’ (10 genes), ‘Immune system’ (8 genes), ‘Signal transduction’ (8 genes), ‘Antigen processing: Ubiquitination and proteasome degradation’ (6 genes), ‘Class I MHC mediated antigen processing and presentation’ (6 genes), ‘Adaptive immune system’ (6 genes), ‘Transmembrane transport of small molecules’ (5 genes) and ‘Gene expression’ (5 genes). Genes involved in major immune pathways were extracted and are listed in Table 8, which shows that 13 genes are linked to 16 pathways with major roles in the innate and acquired immunity as well as cytokine signalling in the immune system.

**Table 7:** List of genes in the significant regions

OAR	Position (Mbp)	Genes
2	106.9–108.4	*GALNTL6*
2	110.1–113.3	*C2H4orf27*, *CLCN3*, *NEK1*, *SH3RF1*, *CBR4*, *PALLD*, *DDX60*, *ANXA10*, *MFSD14B*, *LGSN*, *OCA2*, *HERC2*, *NIPA1*, *NIPA2*, *CYFIP1*, *TUBGCP5*, *PTPN18*, *AMER3*, *ARHGEF4*
2	117.0–118.1	*MAP3K2*, *ERCC3*, *LOC101118856*, *BIN1*, *LOC105608784*, *NAB1*, *NEMP2*, *MFSD6*, *HIBCH*, *INPP1*, *C2H2orf88*, *MSTN*
6	34.7–39.2	*GPRIN3*, *TIGD2*, *FAM13A*, *NAP1L5*, *HERC3*, *PYURF*, *PIGY*, *HERC5*, *HERC6*, *PPM1* *K*, *ABCG2*, *PKD2*, *SPP1*, *MEPE*, *IBSP*, *FAM184B*, *MED28*, *NCAPG*, *DCAF16*, *LCORL*
18	17.6–18.9	*NTRK3*, *DET1*, *MRPS11*, *MRPL46*, *ISG20*, *LOC101123585*
24	40.4–41.9	*IQCE*, *TTYH3*, *LFNG*, *GRIFIN*, *CHST12*, *LOC105604828*, *LOC101112623*, *EIF3B*, *SNX8*, *NUDT1*, *MRM2*, *MAD1L1*, *LOC105604879*, *PSMG3*, *TMEM184A, MAFK*, *INTS1*, *LOC105604880, GPR146*, *C24H7orf50*, *LOC105604882*, *LOC101120042*, *GET4*, *SUN1*, *LOC105604834*, *LOC105604883*, *LOC106991921*, *DNAAF5*, *LOC106991898*, *FAM20C*

**Table 8:** List of genes involved in immune responses and the immune pathways they belong to.

Pathways	OAR2						OAR6					OAR18	
	*SH3RF1*	*HERC2*	*MAP3K2*	*CYFIP1*	*PTPN18*	*BIN1*	*HERC3*	*HERC5*	*HERC6*	*IBSP*	*SPP1*	*ISG20*	*DET1*
Immune system	x	x		x			x	x	x			x	x
Adaptive immune system	x	x					x	x	x				x
Class I MHC mediated antigen processing & presentation	x	x					x	x	x				x
Antigen processing: ubiquitination and proteasome degradation	x	x					x	x	x				x
Innate immune system				x				x					
B cell receptor signaling pathway					x								
Toll-like receptor signaling pathway											x		
Regulation of toll-like receptor signaling pathway											x		
Fc gamma R-mediated phagocytosis						x							
Fcgamma receptor (FCGR) dependent phagocytosis				x									
Interferon signaling								x				x	
Cytokine signaling in immune system								x				x	
Interferon alpha/beta signaling												x	
Antiviral mechanism by IFN-stimulated genes								x					
Interleukin-11 signaling pathway										x			
IL-1 signaling pathway			x										

Ovine chromosomes OAR (*Ovis aries*)

Gene symbols in italics

## Discussion

Our study aimed at detecting genomic regions with effects on parasite resistance in a large population of sheep naturally challenged in the field with mixed parasite species. Both RHM and GWAS identified the region on OAR2 as being significant (FDR of 5%). However, RHM identified additional significant regions at the FDR of 5%, i.e. the regions on OAR6, 18 and 24, which were not detected by GWAS. Both methods use genomic information in different ways, and their power to detect genomic regions depends on the genetic architecture behind the trait. According to Nagamine et al. [16], when trait variation is due to a few causal variants, and those variants are in complete LD with the SNP, then GWAS should be the most powerful approach. However, most complex traits are polygenic with trait variation being explained by variants in many loci, each with a small effect. For such polygenic traits, RHM may be more efficient than the conventional GWAS approach. In principle, RHM facilitates the capture of genetic variance for each region in the genome by integrating the effects of both rare and common variants in a joint analysis. Thus, the RHM approach is potentially capable of identifying loci that cannot be detected by a conventional GWAS analysis. Furthermore, RHM captures the effect of the region, which may also include *cis*-interaction effects between causal genes in that given region. Our results show that some candidate genes within a given region share similar mechanisms related to the immune system, which suggests that some possible interaction effects take place between those genes for protecting the host against parasite infections.

Genomic regions that were identified by RHM as significant explained only a small proportion of the trait variation, ${\text{h}}_{\text{g}}^{2}$ for each region ranged from 0.003 (1.5% of the trait heritability) to 0.01 (5% of the trait heritability), and for all regions combined ${\text{h}}_{\text{g}}^{2}$ was equal to 0.030 (15% of the trait heritability). The small proportion of phenotypic variance explained by the significant regions suggests that parasite resistance is a polygenic trait with a large number of variants involved in the mechanism of resistance. This result is in agreement with Kemper et al. [11], Riggio et al. [12], Riggio et al. [27], and Lee et al. [28] who reported that parasite resistance is a complex trait influenced by a large number of genes each with a relatively small effect. The phenotype as measured in this study was subject to a strict measuring protocol, however, worm egg counts were recorded at different ages and from different parasite species. This likely affects the power and accuracy of the detection of causal variants. However, previous studies have shown that there are high genetic correlations between parasite challenges from different parasite species [29], as well as between WEC measurements at different ages [30].

Regions with a higher impact on parasite resistance were found on OAR2 and OAR6. RHM using 1000-SNP windows identified two regions between 106.4 and 121.1 Mbp on OAR2 and between 30.1 and 42.3 Mbp on OAR6 that were significantly associated with parasite resistance at the genome-wide significance level. Fine-mapping of the region on OAR6 with 200-SNP windows revealed a large region between 35.27 and 38.76 Mbp that passed the genome-wide significance level (FDR ≤ 0.05). Fine-mapping of the region on OAR2 with 200-SNP windows revealed three adjacent regions between 106.9 and 108.4 Mbp, 110.1 and 113.3 Mbp, and 117.0 and 118.1 Mbp that were significant at the genome-wide significance level. Stepwise conditional analyses showed that the proportion of phenotypic variance explained by significant regions gradually increased from 0.4% when regional heritability was based on SNPs from the region between 106.9 and 108.4 Mbp to 0.8% when regional heritability was based on SNPs from all three significant regions on OAR2. These results suggest that several causal mutations are likely responsible for the genetic variation in the OAR2 region.

A comparison with previous studies showed that the region on OAR2 fell within the QTL region (61.7–137.9 Mbp) reported by Crawford et al. [31] for resistance to *T. colubriformis*, which was identified in an outcross of Romney and Coopworth sheep, and which partially overlapped with the QTL region (117.9–133.9 Mbp) reported by Davies et al. [32] for resistance to *Nematodirus spp.* in Scottish blackface sheep. Furthermore, the region on OAR6 has been reported by Riggio et al. [12] for resistance to *Strongyles* in Scottish Blackface sheep. The authors identified a genome-wide significant SNP for *Strongyles* fecal egg count (FEC) in this region on OAR6 using GWAS, and confirmed a QTL region between 33 and 39 Mbp by RHM analysis. The identified region on OAR6 also corresponds to the QTL (25.1-62.6 Mbp) region reported by Silva et al. [10] for resistance to *H. contortus* and *Trichostrongylus spp* in a backcross population of Red Maasai and Dorper sheep. Interestingly, both regions on OAR2 and OAR6 have also been reported as being under selection in a large number of sheep breeds from the Sheep HapMap dataset [33, 34]. RHM analysis also identified a significant region on OAR18 (17.64-18.95 Mbp) and two novel regions between 36.96 and 37.84 Mbp and between 40.47 and 41.99 on OAR24. The identified region on OAR18 partially overlapped with a QTL region identified by Marshal, et al. [35].

*GALNTL6* (*polypeptide N*-*acetylgalactosaminyltransferase*-*like 6*) was the only gene annotated in the region between 106.9 and 108.4 Mbp on OAR2. *GALNTL6* harbours the second and third most significant SNPs detected by GWAS analysis and contains haplotype block 6 (107.33–107.38 Mbp), the only haplotype block identified as having a significant effect on parasite resistance. *GALNTL6* is a member of a highly conserved family of proteins that are responsible for the synthesis of mucin-type O-glycans. Several genes from this family, such as *GALNT1*, *GALNT4* and *GALNT8*, have also been reported as being of importance for sheep resistance to gastrointestinal parasite infections [10, 14, 35]. Abomasum mucus, with mucin as its main component, is considered to be the first line of host defence against invading gastrointestinal parasites [36, 37]. Mucus production during parasite infections is under the immune control of type-2 cytokines [38], with interleukin-4 (IL-4), IL-13, and IL-22 altogether playing the major role in host protection [39–41]. Furthermore, Newlands et al. [42] found that sheep immunized by daily oral challenge with *H. contortus* had unchanged gastric mucin profiles two days after infection, which demonstrates that animals are able to control mucin levels based on their immunological status. In vitro studies showed that parasite feeding and motility are restricted when larvae were co-cultured with sheep intestinal mucus [43].

The region between 110.1 and 113.3 Mbp on OAR2 harbours four genes that are directly involved in immune pathways: *SH3 domain containing ring finger 1* (*SH3RF1*); *E3 ubiquitin*-*protein ligase HERC2* (*HERC2*); *presentation, and cytoplasmic FMR1 interacting protein 1* (*CYFIP1*); and *protein tyrosine phosphatase, non*-*receptor type 18* (*PTPN18*). *SH3RF1* and *HERC2* are involved in the MHC class I mediated antigen processing and presentation pathway. This pathway activates type-1 T lymphocytes (Th1), which is characterized by the production of the cytokine interferon (IFN)-gamma among other cytokines, providing effective cellular response and protection against chronic parasite infection [44]. *CYFIP1* encodes a protein involved in the Fcgamma receptor (FCGR) dependent phagocytosis pathway, a crucial event in the immune system that permits effector cells such as macrophages to uptake and eliminate infectious pathogens. This event is mediated by immunoglobulin (IgG) binding to Fc gamma receptors (Fc gamma R) on the effector cells [45]. *PTPN18* is involved in the B cell receptor signalling pathway, which is essential for the expression of other genes involved with B cell differentiation, proliferation and immunoglobulin (Ig) production.

Other potential genes of interest in this region include: *paladin* (*PALLD*), and *probable ATP*-*dependent RNA helicase DDX60* (*DDX60*). *PALLD* contains the top significant SNPs identified by GWAS analysis. The role of *PALLD* is poorly understood, although it has been found to play an essential role in organizing the skeletal muscle [46]. *DDX60* is important for the production of inflammatory cytokines such as type 1 interferon [47]. In humans, an increased expression of *DDX60* has been detected following viral infections [48, 49], which suggests that *DDX60* is essential to initiate the innate antiviral mechanism.

The region between 117.0 and 118.1 Mbp on OAR2 overlaps with two important genes involved in immune pathways: *bridging integrator 1* (*BIN1*), which encodes a protein being involved in Fc gamma R-mediated phagocytosis pathway, and *mitogen*-*activated protein kinase kinase kinase 2* (*MAP3K2*), which is involved in the IL-1 signalling pathway. *MAP3K2* plays an important role in the IκB kinase (IKK) activation, which is essential for NF-κB (nuclear factor kappa-light-chain-enhancer of activated B cells) signalling [50]. NF-κB regulates the expression of many genes associated with immune responses in the gastrointestinal tract. For instance, NF-κB plays an essential role in the transcriptional regulation of many cytokine genes, including *interferon (IFN)*-*gamma*, *IL*-*1*, *IL*-*2*, *IL*-*6*, and *IL*-*12*, in epithelial cells, lymphocytes and monocytes [51]. The Κ light-chains of NF-κB are also critical components of immunoglobulins, making NF-κB a key regulator of humoral immune responses [52].

The region between 35.27 and 38.76 Mbp on OAR6 overlaps with three genes from the HERC family of ubiquitin ligases (*HERC3*, *HERC5* and *HERC6*) that are associated to four biological pathways (Table 8) including: ‘Immune system’; ‘Antigen processing: Ubiquitination and proteasome degradation’; ‘Class I MHC mediated antigen processing and presentation’; and ‘Adaptive immune system’ pathways.

The OAR6 region also contains two immune genes: *secreted phosphoprotein 1* (*SPP1*) and *integrin binding sialoprotein* (*IBSP*). *SPP1* encodes a protein involved in toll-like receptors (TLR) signalling. TLR are innate immune receptors that detect pathogen invasion in the intestinal mucosa and are essential for mounting a type-2 immune response [53, 54]. This gene also plays an important role in wound healing [54, 55]. *IBSP* is involved in the interleukin-11 (IL-11) signalling pathway. IL-11 accelerates platelet recovery [56], which is important to maintain adequate blood volume levels following parasite infection. Gastrointestinal parasites, especially *H. contortus*, can cause severe blood loss, leading to haemorrhagic anaemia. Maintaining haemostasis is important for sheep recovery following infection as a way to minimise anaemia. Furthermore, haemostasis serves as a defence mechanism to expel parasites from the host body since clotting at the infection site significantly reduces blood supply to adult worms, inhibiting feeding and survival at the infection sites [54].

Other candidate genes in the OAR6 region include: *ligand dependent nuclear receptor corepressor like* (*LCORL*) and *ATP binding cassette subfamily G member 2* (*ABCG2*). *LCORL* contains the sixth most significant SNP detected by GWAS analysis. In most mammalian species, *LCORL* contains trinucleotide repeats in the coding region, resulting in an expanded polyalanine tract in the amino-terminal region of its encoded protein [57]. The extreme expansions of trinucleotide repeats can alter protein function and cause genetic diseases such as the fragile X syndrome and Huntington’s disease [58, 59]. At present, the function and the propensity of repeat expansions in the coding region of the ovine *LOCRL* are not known.

*ABCG2* is highly expressed in the canalicular membrane of the liver, kidney, colon, and in the epithelia of the small intestine [60, 61]. *ABCG2* plays a major role in multidrug resistance [61], and has been identified as a candidate gene for facial eczema in sheep [62]. The expression of *ABCG2* at the apical surface of the intestinal epithelium, a layer of cells that forms a physical barrier between mucosa and the gut luminal content, suggests a potential role for this gene in protecting the host from parasites that try to destroy the lining of the lumen to access the bloodstream.

The region between 17.64 and 18.92 on OAR18 overlaps with the *interferon stimulated exonuclease 20* (*ISG20*) gene, which regulates different cytokine signalling pathways in the immune system including ‘Interferon signalling’ and ‘Interferon alpha/beta signalling’ pathways, and *de*-*etiolated homolog 1* (*DET1*) gene which encodes a protein involved in the ‘class I MHC mediated antigen processing and presentation’ pathway.

Fine mapping RHM analysis using smaller windows identified a novel region on OAR24 between 40.4 and 41.9 Mbp. This region overlaps with the *LFNG O*-*fucosylpeptide 3*-*beta*-*N*-*acetylglucosaminyltransferase* (*LFNG*) gene, which plays an important role in T lymphocyte differentiation and development through regulating notch signalling [63]. The *MAF bZIP transcription factor K* (*MAFK*) gene is another candidate gene in this region that is involved in the haemostasis pathway (see Additional file 1: Table S1), an integral response mechanism against *H. contortus* infection.

## Conclusions

This study identified significant genomic regions on ovine chromosomes 2, 6, 18, and 24 that are associated with parasite resistance in sheep. These results show that RHM is more powerful in detecting regions for parasite resistance and capturing variance than single SNP GWAS. The identified regions overlap with candidate genes that are involved in innate and acquired immune mechanisms, as well as cytokine signalling. Genes involved with haemostasis and mucus production are also relevant for host protection against parasite infections. Our results support the hypothesis that parasite resistance is a complex trait, and is determined by a large number of genes with various roles, rather than by a few genes with a major role in resistance.

## Additional file

**Additional file 1: Table S1.** List of candidate genes and the biological pathways they belong to.
